# Diagnosis and Management of Sarcoidosis-like Reaction in Adjuvant Immunotherapy: A Comprehensive Review and Clinical Implications

**DOI:** 10.3390/biomedicines14051082

**Published:** 2026-05-10

**Authors:** Matthew Lee, Qi Cai, Jue Wang

**Affiliations:** 1 School of Medicine, University of Texas Southwestern Medical Center, Dallas, TX 75390, USA; matthew.lee@utsouthwestern.edu (M.L.); qi.cai@utsouthwestern.edu (Q.C.); 2 Department of Pathology, University of Texas Southwestern Medical Center, Dallas, TX 75390, USA; 3 Harold C. Simmons Comprehensive Cancer Center, University of Texas Southwestern Medical Center, Dallas, TX 75235, USA

**Keywords:** immune checkpoint inhibitors, immune-related adverse events, sarcoidosis-like reaction, adjuvant immunotherapy, granulomatous inflammation, sarcoidosis, mediastinal lymphadenopathy

## Abstract

Immune checkpoint inhibitors (ICIs) have transformed oncologic care and are increasingly used as adjuvant therapy to reduce the risk of recurrence. However, this shift has introduced immune-related adverse events (irAEs) to patients who may otherwise be clinically disease-free after definitive therapy. Sarcoidosis-like reaction (SLR) is an uncommon but important irAE characterized by non-necrotizing granulomatous inflammation. In the adjuvant setting, SLR is uniquely consequential because it can closely mimic recurrent malignancy on surveillance imaging and thereby prompt unnecessary diagnostic procedures, treatment interruption, or escalation of care. This review summarizes the current evidence on ICI-associated SLR with an emphasis on adjuvant immunotherapy, where practical guidance remains limited. We integrate evidence from clinical trials, real-world cohorts, and published case series to summarize the reported incidence of SLR, proposed immunologic mechanisms, clinical and radiographic presentation, pathology, differential diagnosis, and management. Particular attention is given to the problem of distinguishing SLR from recurrence, when tissue confirmation should be prioritized, and how management should be individualized according to clinical severity and organ involvement. Common radiographic features include bilateral mediastinal and hilar lymphadenopathy and pulmonary nodules, but tissue confirmation remains the diagnostic gold standard when feasible. Many cases are low grade and may be managed conservatively. Greater recognition of ICI-associated SLR is critical to avoid misdiagnosis and unnecessary escalation of care while preserving the therapeutic benefit of adjuvant immunotherapy.

## 1. Introduction

Immune checkpoint inhibitors (ICIs) have rapidly expanded from advanced and metastatic disease into earlier-stage and potentially curative settings, including adjuvant therapy [1,2]. In this setting, the goal is to eradicate micrometastatic disease after definitive local therapy and reduce the risk of recurrence [1,2,3,4]. As a result, ICIs are now increasingly used in patients who may otherwise be clinically disease-free. This shift has important implications for how toxicities are interpreted and managed. In metastatic disease, clinicians may accept a higher burden of immune-related adverse events (irAEs) when weighed against meaningful survival benefit [5]. In the adjuvant setting, the risk-benefit balance is more nuanced because patients may be clinically disease-free after local therapy, and even “reversible” toxicities can impair quality of life and delay recovery. Accordingly, patient selection requires careful attention to the baseline recurrence risk and the possibility of clinically meaningful irAEs [6,7].

Sarcoidosis-like reaction (SLR) is a particularly challenging irAE in this setting. SLR is characterized by non-necrotizing granulomatous inflammation that is histologically indistinguishable from sarcoidosis [8]. It typically arises after ICI initiation and may involve mediastinal or hilar lymph nodes, lung parenchyma, skin, and other organs [8,9]. Although SLR is uncommon, its clinical significance extends beyond toxicity alone. In routine practice, it often presents with new thoracic adenopathy, pulmonary nodules, or other FDG-avid lesions on surveillance imaging, creating substantial diagnostic overlap with recurrent malignancy [8,9]. This distinction is consequential in the adjuvant setting, where the central clinical question is often whether new findings represent relapse after curative-intent therapy. Misclassification of SLR may trigger a cascade of inappropriate management decisions, including premature discontinuation of therapy, escalation of systemic treatment, and irreversible local interventions such as stereotactic body radiation therapy (SBRT) or surgery [10].

Despite its clinical importance, the current literature on SLR in adjuvant ICI therapy remains limited. Practical guidance is sparse regarding how to distinguish SLR from recurrence, when biopsy should be prioritized, and how management should be tailored according to diagnostic certainty and organ involvement. In this review, we synthesize the current evidence related to SLR in adjuvant ICI therapy to provide a framework for diagnosis, management, and prevention of overtreatment.

## 2. Pathophysiology and Mechanistic Rationale

The biological basis of ICI-associated SLR remains incompletely defined. Current mechanistic models are derived primarily from preclinical studies, translational immune profiling, and small clinical series rather than prospective investigations [9,11,12,13,14]. One central question is whether ICIs cause de novo sarcoidosis or trigger a drug-induced granulomatous reaction that is histologically indistinguishable from sarcoidosis [9]. Even so, a unifying framework is that checkpoint blockade lowers the threshold for pathologic granulomatous inflammation.

### 2.1. CTLA-4 Pathway

CTLA-4 is an immune checkpoint that limits early T-cell activation in lymph nodes by competing with CD28 for B7 ligands (CD80/86) [15]. CTLA-4 blockade removes this restraint and promotes broader T-cell activation. Downstream, this may favor the same immune architecture observed in sarcoidosis. Melanoma tumor samples obtained after ipilimumab exposure have shown increased interferon gamma (IFN-γ) and type 1 T-helper cell (Th1)-associated immune markers [16]. This is biologically relevant because sarcoidosis has long been considered a Th1-driven disease, and IFN-γ is a key mediator of macrophage activation and granuloma formation [17]. In parallel, sarcoidosis is associated with dysregulation of the T helper 17 (Th17)/regulatory T-cell (Treg) axis, including expansion of Th17 populations [18,19,20]. CTLA-4 blockade has likewise been associated with increases in circulating Th17 cells and a shift in the Th17/Treg balance [11,18]. Together, these observations support a plausible role for CTLA-4 blockade in promoting SLR.

### 2.2. PD-1/PD-L1 Pathway

The PD-1/PD-L1 axis is a central inhibitory checkpoint that restrains peripheral T-cell effector function [21]. Early reports of SLR after PD-1/PD-L1 blockade were viewed as paradoxical because PD-1 signaling appears to be engaged in active sarcoidosis [9]. For example, decreased PD-1 expression has been associated with spontaneous granuloma resolution, and patients with sarcoidosis have increased PD-1 positive CD4^+^ T cells [22]. One proposed mechanism involves Th17.1 cells, which are increasingly recognized as a key CD4^+^ effector in sarcoidosis [12,20]. In a melanoma case series, patients who developed ICI-associated SLR were found to have elevated baseline Th17.1, suggesting PD-1 blockade may amplify Th17.1 responses [23].

A second, more downstream mechanism may involve metabolic support of granuloma persistence through the phosphoinositide 3-kinase (PI3K)-protein kinase B (Akt)-mechanistic target of rapamycin (mTOR) signaling axis. PD-1 signaling normally suppresses PI3K activation and downstream Akt phosphorylation, which limits mTOR pathway activation [24]. By releasing this restraint, PD-1/PD-L1 blockade may enhance PI3K-Akt-mTOR signaling, which is a pathway implicated in sarcoidosis pathogenesis [14]. Linke et al. demonstrated that constitutive activation of mTORC1 in myeloid cells was sufficient to drive granuloma formation in vivo. They also found that pharmacological mTOR inhibition promoted granuloma resolution [25]. Accordingly, PD-1/PD-L1 inhibition may contribute to SLR through two complementary effects: amplification of pathogenic T-cell effector populations (i.e., Th17.1) and enhancement of PI3K-Akt-mTOR signaling that supports granuloma maintenance.

### 2.3. Integrated Mechanistic Model

Overall, the available evidence supports a model where checkpoint blockade creates a permissive environment for non-necrotizing granuloma formation. ICIs release the inhibitory restraint on T cells, leading to enhanced T-cell activation; this favors Th1/Th17.1-skewed inflammation and impaired regulatory control. These changes promote macrophage recruitment and activation, epithelioid histiocyte transformation, and multinucleated giant cell formation, resulting in the development and persistence of non-necrotizing granulomas [9,11,12,13,14]. Within this sequence, CTLA-4 blockade may contribute to the initiation of granulomatous inflammation by promoting early T-cell priming and weakening regulatory control. Conversely, PD-1/PD-L1 blockade may amplify peripheral effector responses and help sustain granulomatous inflammation through PI3K-Akt-mTOR signaling. Although the precise biology remains incompletely defined, these convergent observations support a mechanistic framework for ICI-associated SLR [9,11,12,13,14].

## 3. Epidemiology and Evidence in Adjuvant Therapy

The true incidence of ICI-associated SLR is difficult to estimate because detection depends on surveillance intensity, coding of adverse events, study design, and whether tissue confirmation is pursued. The estimated incidence ranges from <0.5% in large metastatic clinical trial datasets to over 20% in real-world adjuvant cohorts [1,10,26].

Across tumor types, SLR is most commonly reported in melanoma [8,9,26]. In metastatic/advanced disease, SLR is an uncommon event, and it is rarely listed as a discrete adverse event in phase III trial safety tables (e.g., CheckMate 067) [27]. In a large single-center cohort spanning multiple tumor types (2963 ICI-treated patients), the overall incidence of SLR was 0.24% [26]. Similarly, SLR was reported in <0.5% of patients (1/207) in an early anti-PD-L1 dataset and 0.2% (2/908) in a registry of anti-PD-1/PD-L1-treated patients [8,28]. The contrast with adjuvant cohorts is striking. In the phase III KEYNOTE-054 adjuvant melanoma trial, sarcoidosis occurred in 1.4% of pembrolizumab recipients [1]. By comparison, in a real-world adjuvant melanoma cohort (nivolumab ± ipilimumab), Chorti et al. observed SLR in 22% of patients (10/45). These patients were largely asymptomatic, detected on scheduled imaging, and histologically confirmed [10].

The most plausible explanation for this discrepancy is ascertainment bias. In metastatic disease, new thoracic adenopathy or pulmonary nodules may be attributed to progressive disease or nonspecific inflammation, and biopsy could be deferred when the result is unlikely to alter management [8]. In addition, if SLR is not prespecified in adverse event reporting or is coded under broader categories (e.g., lymphadenopathy), cases may go unrecognized in trial datasets. Accordingly, although most metastatic series suggest an incidence below 1%, these estimates likely underestimate the true frequency of SLR. By contrast, adjuvant cohorts often undergo protocolized surveillance imaging in patients without measurable disease. Clinicians may have a lower threshold to biopsy equivocal findings because the implications of mislabeling recurrence are considerable. A true biological contribution cannot be excluded, although the rates of irAEs appear to be similar in adjuvant versus advanced melanoma cohorts [29]. At present, the available evidence more strongly supports ascertainment bias and diagnostic intensity, rather than biological differences, as the main drivers of the reported incidence discrepancy.

## 4. Clinical Spectrum and Imaging Features

### 4.1. Timing of Onset

ICI-associated SLR typically develops early after treatment initiation, most commonly between the first 3–6 months of therapy [8,9,10,30,31]. In an adjuvant melanoma cohort, SLR was usually detected at the first surveillance scans with a median time of 2.8 months [10]. However, cases of SLR have been described as early as 3 weeks and as late as 2 years after initiation of ICIs [9]. SLR has even been identified following ICI discontinuation, underscoring the need for ongoing vigilance during follow-up [10].

### 4.2. Clinical Presentation & Organ Distribution

SLR is most often a low-grade irAE (typically grade 1–2), and clinically significant organ dysfunction is uncommon in most series [1,10]. In KEYNOTE-054, all reported cases of SLR were grade 1 or 2 [1]. However, symptomatic presentations do occur, and one series reported 92% (11/12) of patients experienced symptoms [32]. Similar to sarcoidosis, the lungs and intrathoracic lymph nodes are by far the most frequent site [9,10,30,33]. In one series, pulmonary involvement was reported in 85% of patients (50/59) [30]. When SLR is symptomatic, respiratory symptoms (cough, dyspnea) are the most common [32]. Patients may also experience constitutional symptoms (e.g., fevers, night sweats) [32].

Beyond thoracic involvement, SLR has been shown to affect virtually any organ system [33]. Cutaneous disease is the most common extrathoracic manifestation and is seen in up to ~50% of patients in pooled series [8,30]. Clinically, this may present as subcutaneous nodules, erythematous papules or plaques, and even granulomatous inflammation arising within scars or tattoos [30,34]. Ocular involvement (e.g., dry eye syndrome, acute iritis, uveitis) is less common but important given the potential for vision-threatening complications [14,34]. Other reported sites include the liver, spleen, peripheral lymph nodes, kidney, and bone [8,30,34]. While uncommon, these patterns can closely mimic metastatic disease and may occasionally present as organ dysfunction such as an acute kidney injury in renal SLR [35]. Rare neurologic or cardiac involvement has also been described, and these presentations can be clinically severe and warrant prompt recognition and treatment [36,37].

### 4.3. Imaging Features & Supportive Laboratory Findings

**Figure 1:**
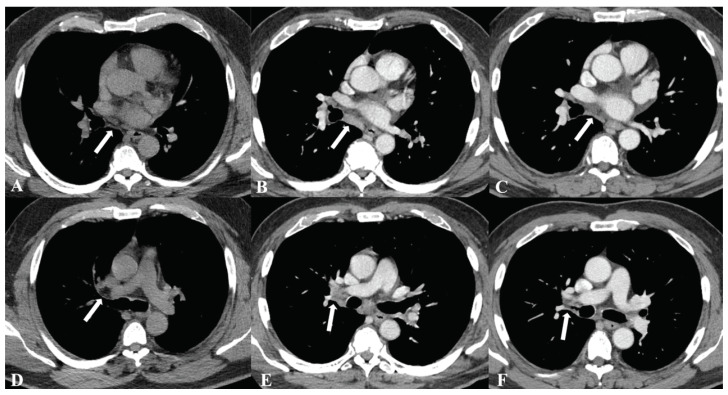
Biopsy-proven immune checkpoint inhibitor (ICI)-associated sarcoidosis-like reaction (SLR) on serial CT imaging in a patient with renal cell carcinoma (RCC) treated with adjuvant pembrolizumab. Images are derived from a previously reported case from our group [31]; distinct radiologic images are presented here to avoid duplication and to illustrate characteristic findings and temporal evolution during ICI therapy. (**A**–**C**) Subcarinal lymph node (arrow): (**A**) baseline noncontrast CT shows no adenopathy; (**B**) interval development of a 10 mm subcarinal lymph node on contrast-enhanced CT; (**C**) regression after corticosteroids with no residual adenopathy by CT size criteria. (**D**–**F**) Right hilar lymph node (arrow): (**D**) baseline noncontrast CT shows no adenopathy; (**E**) interval development of a 14 mm hilar lymph node on contrast-enhanced CT; (**F**) regression after corticosteroid therapy with no residual adenopathy by CT size criteria.

ICI-associated SLR is often first suspected on restaging or surveillance imaging, and its radiographic features mirror sarcoidosis (Figure 1). On CT, bilateral mediastinal and hilar lymphadenopathy is considered the classic radiographic feature of sarcoidosis [38]. In a large registry study, 100% of patients (32/32) with ICI-associated SLR demonstrated bilateral hilar lymphadenopathy, underscoring how SLR follows the typical pattern of sarcoidosis [39]. High-resolution CT may show a perilymphatic distribution of micronodules, a classic pattern of sarcoidosis [38,40]. Certain CT patterns such as symmetric hilar involvement can raise suspicion for a granulomatous process [38]. However, in adjuvant surveillance, the clinical significance of these findings is amplified because new intrathoracic adenopathy may be interpreted through a recurrence-focused lens.

On FDG-PET scans, ICI-associated SLR is typically FDG-avid, with uptake in involved lymph nodes and organs that can be indistinguishable from malignancy [8]. This parallels sarcoidosis, where granulomatous inflammation is often intensely hypermetabolic [41]. While the most frequent PET pattern is bilateral mediastinal and hilar nodal uptake, PET/CT can also reveal a broader distribution and complicate staging [14]. For instance, intrasplenic and osseous FDG-avid lesions have been described, which mimic metastatic disease [8,42]. Thus, imaging can raise suspicion for SLR and help define disease extent or identify a biopsy target, but it cannot reliably distinguish SLR from recurrence or infection.

Supportive laboratory findings are similarly nonspecific. Angiotensin-converting enzyme (ACE) levels may be elevated in patients with SLR [8]. In a series of patients with SLR, 67% of patients (4/6) had elevated ACE [32]. However, ACE has an estimated sensitivity of ~41% for sarcoidosis, and cases of SLR have documented normal values of ACE [31,43]. Compared to ACE, soluble interleukin-2 receptor (sIL-2R) appears to be more robust with a sensitivity of ~88% for sarcoidosis [44]. A higher sIL-2R level is associated with more extensive pulmonary involvement in sarcoidosis; so, it may be a helpful clue for diagnosis [44]. Hypercalcemia is occasionally seen in sarcoidosis due to calcitriol production in granulomas, and it has been documented in patients with ICI-associated SLR [32]. Overall, laboratory findings of SLR largely mirror those of sarcoidosis and are supportive rather than diagnostic.

## 5. Differential Diagnosis & Diagnostic Approach

### 5.1. Differential Diagnosis

In adjuvant patients, new FDG-avid lymphadenopathy or pulmonary nodules should be treated as a high-stakes diagnostic problem [9,13]. In this context, the practical differential diagnosis for a sarcoid-like radiographic pattern on ICIs includes: (1) recurrent/progressive malignancy, (2) granulomatous infection (e.g., mycobacterial and fungal infections), (3) ICI-associated SLR, (4) other noninfectious inflammatory etiologies (e.g., granulomatosis with polyangiitis), and less commonly (5) lymphoma or tumor-associated granulomas (Table 1) [9].

ICI-associated SLR is most strongly suggested by symmetric bilateral hilar and mediastinal lymphadenopathy, particularly when these findings arise after ICI initiation in otherwise controlled disease [38,39]. Tumor recurrence or progression is more likely when imaging shows asymmetric or progressively enlarging lymphadenopathy, new solid-organ lesions, or a distribution typical of metastatic spread of the underlying cancer [45]. Granulomatous infection, including mycobacterial and fungal disease, should be considered when there are necrotic nodes, cavitary nodules, tree-in-bud opacities, or relevant exposure or endemic risk factors [45,46].

### 5.2. Diagnostic Approach

The diagnosis of SLR follows the core principles of sarcoidosis: (1) a compatible clinical and radiologic presentation, (2) pathologic evidence of noncaseating granulomas, and (3) exclusion of alternative causes of similar findings [47]. In adjuvant oncology, tissue confirmation should be strongly considered when the diagnosis is uncertain, when recurrence cannot be confidently excluded, or when the result would alter oncologic management. However, biopsy may not be required in every case. In carefully selected patients with a highly characteristic radiographic pattern, low suspicion for recurrence, multidisciplinary consensus, and no organ-threatening involvement, a diagnosis based on imaging and clinical features with close interval follow-up is reasonable; however, such a diagnosis should remain provisional and accompanied by close interval reassessment. Progression on follow-up, emergence of atypical features, or development of symptoms should prompt renewed evaluation for recurrence, infection, or alternative inflammatory disease. Importantly, biopsy is commonly performed and decisive in patients with SLR [9,10]. For instance, in an adjuvant melanoma cohort, biopsies were obtained in 8/10 patients, which confirmed SLR, underscoring the central role of tissue confirmation when there is diagnostic uncertainty [10].

When tissue confirmation is pursued, the biopsy target should be chosen on the basis of safety, accessibility, and diagnostic yield. Given the frequency of cutaneous involvement, superficial lesions such as skin or subcutaneous nodules should be sampled when present [48]. In patients with predominantly intrathoracic lymphadenopathy, endobronchial ultrasound-guided transbronchial needle aspiration of the mediastinal/hilar lymph nodes is often the most practical and highest-yield approach, with diagnostic performance exceeding 90% in sarcoidosis series [31,49,50].

**Figure 2:**
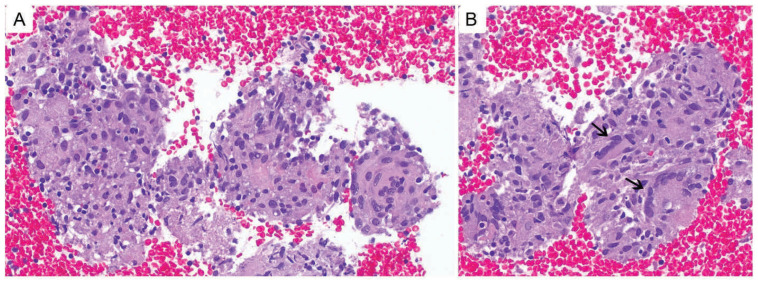
Biopsy-proven immune checkpoint inhibitor (ICI)-associated sarcoidosis-like reaction (SLR) in a patient with renal cell carcinoma (RCC) treated with adjuvant pembrolizumab. Histopathologic images are derived from a previously reported case from our group [31] and show distinct pathology images selected to avoid duplication while illustrating characteristic granulomatous findings during ICI therapy. (**A**) Low-power view showing a discrete non-necrotizing granuloma composed of tightly compact epithelioid histiocytes with abundant eosinophilic cytoplasm. (**B**) Higher-power view showing multinucleated giant cells (black arrows) within the granuloma.

**Figure 3:**
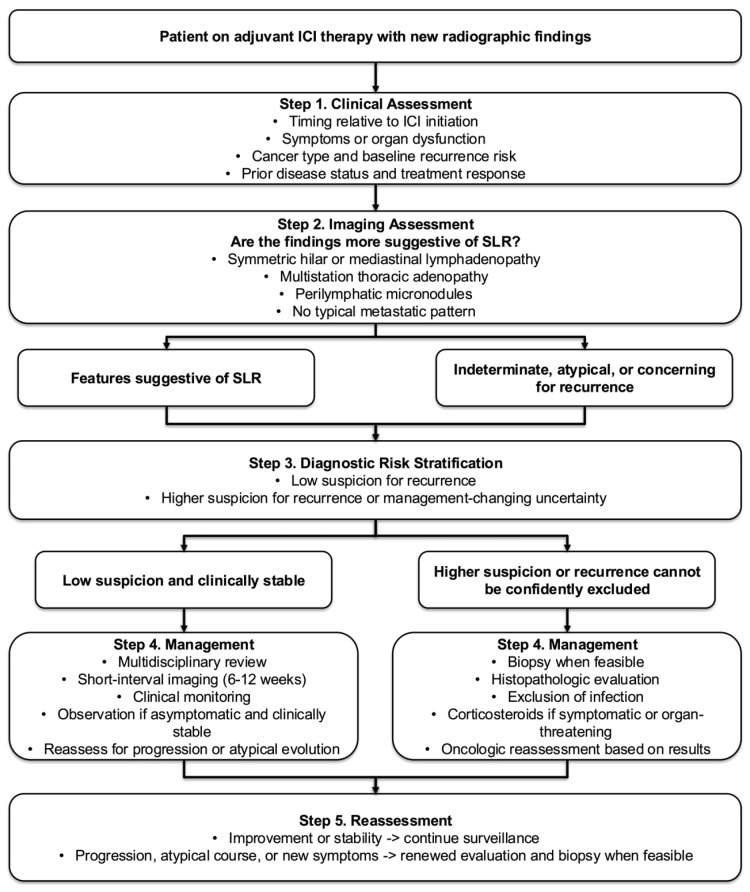
Proposed risk-adapted diagnostic approach for suspected ICI-associated SLR in adjuvant therapy. This schematic outlines a risk-adapted diagnostic approach to differentiate ICI-associated sarcoidosis-like reaction from tumor recurrence. The algorithm integrates clinical context, imaging features, and diagnostic risk stratification to guide decisions regarding close interval follow-up versus biopsy when feasible. This framework aims to balance diagnostic uncertainty with avoidance of unnecessary invasive procedures and inappropriate treatment changes.

In pathology, the demonstration of noncaseating granulomas with no evidence of tumor (Figure 2) is consistent with SLR in the right context [8]. However, mycobacterial and fungal infection remain among the most common causes of granulomatous inflammation in the lung, and tuberculosis may present with noncaseating granulomas [9,51]. Histopathology can therefore help rule out malignancy, but it cannot by itself establish the diagnosis of SLR. This distinction is particularly important in patients with cancer, who may be immunocompromised and are at higher risk for opportunistic infections [52]. Accordingly, guidelines recommend AFB staining and latent TB testing as a standard approach in suspected sarcoidosis [47]. Reflecting this principle, many published ICI-associated SLR cases explicitly document negative infectious studies before assigning the diagnosis [8,31,49]. When feasible, exclusion of malignancy and infection should precede the diagnosis of SLR and any major oncologic decision-making in adjuvant settings (Figure 3).

## 6. Management Strategies

### 6.1. Initial Management in Adjuvant Therapy

Once the diagnosis is established, management should be driven by symptom burden, organ threat, and the oncologic value of continuing adjuvant therapy. Current ASCO irAE guidelines do not provide a dedicated algorithm for SLR, but they support the continuation of ICIs for many grade 1, non-organ threatening toxicities [53]. Within this framework, SLR may not require ICI discontinuation if symptoms are absent or mild and no organ-threatening involvement is present. This approach is supported by cohort data [8,10,39]. In the largest biopsy proven cohort to date (32 cases), there were no severe manifestations, and only 37% of patients (12/32) required systemic therapy [39]. In an adjuvant melanoma cohort, 70% of patients (7/10) with SLR continued ICI therapy, and no patients required systemic steroids [10]. In a mixed tumor cohort, only 14% of patients (1/7) who continued ICI therapy experienced a second flare of SLR. The authors concluded that ICI-associated SLR does not necessarily require immunotherapy discontinuation [39]. Accordingly, the continuation of ICI therapy with close monitoring is reasonable when SLR is asymptomatic or mildly symptomatic, confined to noncritical organs, and not associated with organ-threatening involvement [39,54].

### 6.2. Escalation Therapy & Organ-Specific Considerations

Because there is no universally adopted SLR-specific protocol, management typically follows consensus irAE guidance. For grade 2 toxicities, ICIs are often held and oral prednisone (~0.5–1 mg/kg/day) is initiated, with a taper as symptoms improve. For grade 3 toxicities, ICIs are held and high-dose corticosteroids (prednisone 1–2 mg/kg/day or equivalent) are recommended, with a taper over at least 4–6 weeks [53]. Therefore, SLR is treated based on symptom burden and organ threat rather than radiographic extent alone.

Published cohorts highlight that ICI interruption and steroids are common in practice (Table 2). In a mixed tumor cohort of 32 SLR cases, ICIs were held in 78% of patients (25/32) and were ultimately definitively discontinued in 56% (18/32) [39]. In a melanoma-focused review, Melin et al. reported steroids in 37% of cases (25/69) and ICI discontinuation in 49% of published cases (33/67) [14]. Finally, in an institutional series, ICIs were permanently discontinued in 58% of patients (7/12), most commonly for pulmonary symptoms, whereas 42% of patients (5/12) continued ICI therapy after SLR diagnosis [32]. Importantly, these relatively high discontinuation rates should not be interpreted to mean that SLR mandates treatment cessation. Many reported cohorts were drawn largely from metastatic populations and are likely subject to ascertainment bias, as asymptomatic adenopathy or pulmonary nodules may be attributed to background inflammatory change or disease progression in this setting [14,39]. In many cohorts, treatment was held or discontinued in patients with symptomatic pulmonary involvement, multisystem disease, or concurrent irAEs that prompted a more cautious management approach [14,26,32]. Additionally, treatment interruption or discontinuation may reflect clinician judgment that the anticipated benefit of continuing immunotherapy is limited, particularly when the patient has already demonstrated meaningful oncologic benefit [32].

An additional management consideration is the potential impact of corticosteroids on antitumor immunity. Although steroids can theoretically counteract the immune activation required for ICI efficacy, the literature has been mixed [55]. Importantly, much of the observed association between steroid exposure and inferior oncologic outcomes derives from baseline or early corticosteroid use [55,56]. In contrast, the effect of corticosteroids administered for irAE management is less certain and potentially confounded by treatment indication, timing, and toxicity severity [55,57]. Thus, corticosteroids should not be withheld when SLR is symptomatic or organ-threatening, but treatment should generally follow the lowest effective dose and shortest effective taper needed to achieve control [53].

Truly steroid-refractory SLR is uncommon. As Gkiozos et al. noted, most cases will resolve with discontinuation of ICIs and/or steroid use [8]. Steroid-sparing immunosuppression should be reserved for rare steroid-refractory SLR or organ-threatening presentations, ideally after multidisciplinary discussion. In one review, methotrexate and infliximab were rarely used to manage SLR (2.4% and 3.5%, respectively) [58]. These agents have been described in more severe phenotypes, such as neurosarcoidosis-like presentations [34].

Management should also be individualized by organ system. For cutaneous SLR, local therapy is often sufficient, and topical (or intralesional) corticosteroids have been used successfully [30,49]. Ocular SLR requires early ophthalmology involvement given the risk of vision-threatening complications, and reported cases have been managed with topical steroid eye drops, with escalation to systemic corticosteroids for more severe disease [34]. Rare phenotypes such as renal, neurologic, and cardiac SLR should be approached as potentially organ-threatening irAEs. In these cases, ICIs should be held, high-dose systemic corticosteroids should be started promptly, and multidisciplinary care should be arranged early. If there is inadequate improvement within 48–72 h, second-line immunosuppression may be required in accordance with established irAE guidelines [35,48,53].

## 7. Discussion

The migration of ICIs into adjuvant therapy represents a double-edged sword in modern oncology. These agents can meaningfully reduce recurrence risk in high-risk patients. However, they may also induce toxicities in patients who are clinically disease-free after definitive local therapy, amplifying the downstream consequences of adverse events [1,2,4]. SLR underscores this tension because it can resemble the diagnosis that matters the most in surveillance. In high-risk patients, that resemblance can prompt escalation before the diagnosis is confirmed [10,32].

One unresolved question is whether ICI-associated SLR may serve as a biomarker for antitumor activity. More broadly, the development of certain irAEs, particularly for cutaneous and some endocrine toxicities, is associated with improved outcomes during ICI therapy [5,59]. Several retrospective series have reported that SLR often occurs in patients with concurrent disease control or favorable oncologic status, suggesting that granulomatous inflammation may reflect robust immune activation [10,14,39]. This observation is consistent with the proposed mechanism that checkpoint blockade may lower the threshold for granulomatous inflammation by augmenting antitumor immune activation, although the precise biology remains incompletely defined. Reflecting this, Nykaza et al. found that 92% (11/12) of patients with SLR had radiographic benefit from ICIs, and Chorti et al. found that most patients remained relapse-free on follow-up [10,32]. However, current evidence is limited by small sample size and retrospective design. At present, SLR should not be regarded as a validated biomarker of therapeutic response, but future prospective testing is warranted.

Although organ dysfunction is uncommon in aggregate cohorts, SLR is not solely a mimic of recurrence. Symptomatic burden can be clinically meaningful and is frequently reported [32]. In the adjuvant setting, where quality of life and recovery after local therapy are central, the harms may extend beyond physiologic toxicity. Imaging itself can be a source of substantial distress and anxiety, especially when SLR creates equivocal radiographic findings [60]. In addition, repeated CT/PET-based workups increase cumulative radiation exposure and contribute to financial stressors [61,62]. The risk–benefit shift is magnified in older adults and frail patients who are increasingly treated with ICIs in routine practice [63]. Those with frailty and geriatric impairments may have worse outcomes, increased hospitalization, or experience functional decline from irAEs [63,64,65]. In this context, even a low-grade SLR can be consequential if it triggers invasive workups, anxiety, or steroid exposure that accelerates deconditioning.

A special scenario is pre-existing sarcoidosis because it can blur the line between a new irAE and reactivation. In the largest published cohort of patients with a history of sarcoidosis treated with ICIs (n = 32), only one patient (3%) had a sarcoidosis flare. This patient experienced a major flare involving multiple organs and required steroids and eventually low-dose methotrexate [66]. In another cohort of 12 patients with ICI-associated SLR, 1 patient (8%) had a pre-existing diagnosis of sarcoidosis. This patient suffered from an acute kidney injury and was treated with steroids [32]. These observations suggest that flares are uncommon but can be clinically consequential. Additionally, SLR may present alongside other irAEs [32]. When this occurs, the most clinically urgent toxicity dictates therapy, although immunosuppression may obscure the natural history and diagnostic interpretation of granulomatous findings [32,53]. Early multidisciplinary coordination is especially important in adjuvant surveillance settings to avoid premature recurrence labeling while ensuring organ-threatening irAEs are promptly treated.

## 8. Conclusions

SLR is a uniquely consequential irAE in adjuvant immunotherapy settings because it can closely mimic recurrence in patients who may otherwise be clinically disease-free. In this context, the greatest risk may not be SLR itself, but the downstream cascade triggered by misdiagnosis, including premature treatment discontinuation, systemic treatment escalation, or irreversible local therapy that may ultimately harm the patient. New FDG-avid lymphadenopathy or pulmonary findings during adjuvant therapy should not be presumed to represent recurrence without careful evaluation. Before formally diagnosing SLR, malignancy and infection should be reasonably excluded, and tissue confirmation should be pursued when feasible, particularly when the diagnosis would alter oncologic management. Once SLR is established, management should be individualized according to symptom burden, organ threat, and the oncologic value of continuing ICI therapy. Accordingly, a multidisciplinary approach offers the best opportunity to prevent overtreatment, preserve quality of life, and maintain the therapeutic benefit of ICIs in adjuvant settings.

## 9. Future Directions

Future progress in SLR should prioritize reducing diagnostic ambiguity, refining risk stratification, and standardizing management. First, larger prospective studies, pooled analyses, and studies with standardized surveillance protocols, biopsy practices, and case definitions are needed to define the true incidence, clinical spectrum, and natural history of SLR in adjuvant populations. Second, SLR needs validated adjunctive tools to help distinguish SLR from recurrence. One potential opportunity is to integrate minimal residual disease biomarkers into diagnostic pathways. For instance, circulating tumor DNA (ctDNA) has been shown to predict relapse risk and enable relapse monitoring after definitive local therapy in multiple cancers [67,68,69]. Although ctDNA is not validated to distinguish SLR from recurrence, it may be a useful tool in select malignancies when surveillance imaging is equivocal. In practical terms, a newly positive or rising ctDNA in a clinically stable patient with indeterminate new adenopathy or inflammatory-appearing lesions could justify lowering the threshold for biopsy, PET/CT, or other recurrence-directed evaluation. For example, a rising ctDNA level in a patient with melanoma could justify lowering the threshold for more aggressive recurrence workup [69]. Conversely, persistently negative ctDNA might support short-interval repeat imaging while tissue confirmation is still pursued when feasible. Importantly, ctDNA should only be used as an adjunct to clinical assessment and imaging and not as a substitute for biopsy. Prospective studies are needed to determine how ctDNA can be integrated into real-world diagnostic algorithms. Additionally, radiomics and machine learning represent promising strategies to reduce diagnostic uncertainty from imaging. Proof-of-concept studies have shown that sarcoidosis and lymphoma can be differentiated using machine learning and radiomics, even when human interpretation is uncertain [70]. CT-based radiomics has also been reported to discriminate mediastinal lymphadenopathy from sarcoidosis versus lymphoma with high performance [71]. While these tools have not been validated to distinguish ICI-associated SLR from recurrence, they may eventually help guide clinical decision-making. Finally, consensus frameworks are needed to guide corticosteroid initiation, immunotherapy interruption or rechallenge, and organ-specific management beyond broad irAE guidelines. These advances will help minimize overtreatment and support safer integration of ICIs into adjuvant settings.

## Figures and Tables

**Table 1:** Differential diagnosis of a “sarcoid-like” pattern during ICI therapy.

Competing Diagnosis	Imaging Clues	Temporal Evolution	Clinical Features
ICI-Associated SLR	Symmetric hilar/mediastinal lymphadenopathy ± perilymphatic lung nodules	Often develops within the first few months after ICI initiation; may stabilize or regress with ICI interruption	May be asymptomatic; cutaneous or extrapulmonary granulomatous findings may coexist; ACE, sIL-2R, or calcium may be abnormal
Recurrent/Progressive Malignancy	Progressive enlargement on serial imaging; typical metastatic pattern for the tumor; asymmetric nodal disease	Progressive enlargement on serial imaging; may coincide with progression at other known sites	Rising tumor marker when applicable; high recurrence risk
Granulomatous Infection (Mycobacterial or Fungal)	Nodules ± cavitation; necrotic lymph nodes; tree-in-bud pattern	Variable; may evolve despite ICI interruption	Exposure/endemic risk; immunosuppression; B symptoms; microbiologic studies (e.g., AFB stain, culture, PCR, fungal testing)
Lymphoma/Tumor-Associated Granulomas	Bulky/asymmetric lymphadenopathy; atypical distribution; rapid nodal progression	Progressive nodal enlargement	Generalized or atypical adenopathy; cytopenia; B symptoms
Other Inflammatory Etiologies (e.g., GPA/vasculitis)	Multiple pulmonary nodules ± cavitation	Depends on underlying disease process	ENT symptoms or hematuria/proteinuria; systemic vascular features (e.g., purpura or mononeuritis multiplex)

This table summarizes imaging, temporal, and clinical features that may help distinguish ICI-associated sarcoidosis-like reaction from recurrent malignancy, granulomatous infection, lymphoma or tumor-associated granulomas, and other inflammatory etiologies. Abbreviations: ENT, ear/nose/throat; GPA, granulomatosis with polyangiitis; ICI, immune checkpoint inhibitor; SLR, sarcoidosis-like reaction; TB, tuberculosis.

**Table 2:** Selected studies on the diagnosis and management of ICI-associated SLR.

Study	Cancer Type	Treatment Setting	SLR Cases (*n*)	Diagnostic Confirmation	Management ^a^	Outcome of SLR ^b^
Chorti, 2020 [10]	Melanoma	Adjuvant	10	Biopsy 8/10	ICI continued 7/10; ICI discontinued 3/10	Resolved/improved 10/10
Chanson, 2021 [39]	Mixed solid tumors	Mixed (mostly metastatic)	32	Biopsy 32/32	ICI held 25/32; ICI permanently discontinued 18/32; steroids 9/32	CR/PR 21/25; relapse after rechallenge 1/7
Melin, 2022 [14]	Melanoma	Mixed (mostly metastatic)	18	Biopsy 18/18	ICI discontinued 7/18; steroids 7/18	Clinical/biologic regression 18/18
Smith, 2024 [26]	Melanoma and NSCLC	Mixed (mostly advanced/metastatic)	7	Biopsy 7/7	ICI continued 3/7; steroids + permanent ICI discontinuation 2/7	NR
Nykaza, 2025 [32]	Mixed solid tumors	Advanced/metastatic	12	Biopsy 12/12	ICI continued 5/12; ICI permanently discontinued 7/12; steroids 7/12; hydroxychloroquine 1/12	Improved with treatment in most cases (not quantified)

This table summarizes key published cohorts, including treatment setting, diagnostic confirmation, management approach, and reported outcomes of sarcoidosis-like reaction. ^a^ Held = temporary interruption & resumed; discontinued = stopped (temporary vs. permanent not specified); permanently discontinued = explicitly not restarted ^b^ Response and survival outcomes are reported as described in the original studies and were not uniformly defined across cohorts. Abbreviations: CR, complete response; ICI, immune checkpoint inhibitor; NR, not reported; NSCLC, non-small cell lung cancer; PR, partial response; SLR, sarcoidosis-like reaction.

## Data Availability

The original contributions presented in this study are included in the article. Further inquiries can be directed to the corresponding author.
